# Profiles of volatile sulfur compounds in various vegetables consumed in Korea using HS-SPME-GC/MS technique

**DOI:** 10.3389/fnut.2024.1409008

**Published:** 2024-07-22

**Authors:** Samuel Park, Heon-Woong Kim, Chang Joo Lee, Younghwa Kim, Jeehye Sung

**Affiliations:** ^1^Department of Food Science and Biotechnology, Andong National University, Andong, Gyeongbuk, Republic of Korea; ^2^National Institute of Agricultural Sciences, Rural Development Administration, Wanju-gun, Jeonbuk, Republic of Korea; ^3^Department of Food Science and Biotechnology, Wonkwang University, Iksan, Jeonbuk, Republic of Korea; ^4^Department of Food Science and Biotechnology, Kyungsung University, Busan, Republic of Korea

**Keywords:** volatile sulfur compound, HS-SPME/GC–MS, method validation, vegetables, chemometric analysis

## Abstract

Volatile sulfur compounds (VSCs) are not only important for their therapeutic potential but also significantly influence the flavor profiles of agricultural products. VSCs exhibit various chemical structures due to their stability and volatility, and they may form or be altered as a result of enzymatic and chemical reactions during storage and cooking. This study has focused on profiles of VSCs in 58 different vegetable samples by using HS-SPME-GC/MS technique and chemometric analyses. The validation was carried out using cabbage juice as a vegetable matrix for VSCs analysis, showing satisfactory repeatability (RSD 8.07% ~ 9.45%), reproducibility (RSD 4.22% ~ 7.71%), accuracy and specificity. The established method was utilized on various vegetables, revealing that 21 VSCs such as sulfides, disulfides, trisulfides, isothiocyanates, sulfhydryls, and thiophenes were successfully identified and quantified. These compounds were found in a range of vegetables including *Allium* species, *Cruciferae*, *Capsicum* species, green leafy vegetables, and mushrooms. In particular, isocyanate and allyl groups were abundant in *Cruciferae* and *Allium* vegetables, respectively. Cooking conditions were shown to reduce the levels of certain sulfur compounds such as dimethyl sulfide and dimethyl trisulfide in vegetables like broccoli and cabbage, suggesting that heat treatment can lead to the volatilization and reduction of these compounds. The present study provides reliable insights into the compositions of VSCs in various vegetables and examines the changes induced by different cooking methods.

## 1 Introduction

Sulfur is essential for the synthesis of metabolites related with central roles in metabolic health and homeostasis (1). Dietary sulfur-containing compounds, are widely distributed in vegetable matrices such as *Allium* species and *Cruciferae*, contributing not only to various health effects such as antioxidant, anti-cancer, and anti-inflammatory properties but also to strong aromas and spicy flavors (1, 2). These are classified into non-volatile compounds (i.e., glucosinolates) and volatile compounds (i.e., sulfides, isothiocyanates, sulfhydryls, and thiophenes). Glucosinolates are relatively stable compounds; however, plant cells containing them can be easily damaged, leading to the release of β-thioglucosidase or myrosinase enzymes during the cutting, mixing, chopping, and chewing of food, which produces volatile sulfur compounds (VSCs) (3, 4). VSCs might be the major form of sulfur-containing secondary metabolites in vegetables due to enzymatic and chemical transformations during food processing, cooking and storage periods in vegetables, even though sulfur-containing compounds are classified into glucosinolates, sulfides, isothiocyanates, sulfhydryls, and thiophenes (5). Many studies have reported that VSCs possess beneficial effects such as anti-inflammatory, antioxidant, anti-cancer, cholesterol-lowering, and improvement of atherosclerosis (6–9). Particularly, *Allium* vegetables, such as garlic, have been recommended as a nutritional supplement to prevent cardiovascular disease with a reduced risk and incidence of atherogenesis (10, 11). Consumption of *Cruciferae* vegetables (i.e., broccoli family) is also associated with a lower risk of several cancers and a reduction of systemic inflammation (12).

The major VCSs of *Allium* vegetables have been identified as allicin, diallyl trisulfide (DATS), diallyl disulfide (DADS), and *S*-allyl mercaptan (SAMC), which can be converted from the non-volatile precursor (i.e., alliin) by endogenous alliinase (13). In *Cruciferae* vegetables, the glucosinolate precursor is hydrolyzed by endogenous myrosinase, cleaving the β-thioglucose bond in glucosinolates, resulting in the formation of diverse isothiocyanates (ITCs) (14). These enzymatic reactions are inevitable during the chewing of vegetables as well as the culinary actions such as cutting, mixing, chopping, and heating (15, 16). Even though sulfur-containing amino acids (i.e., tripeptides and glutathione) are the predominant dietary forms of sulfur compounds, plant-derived sulfur-containing secondary metabolites, such as VSCs, might play important roles in antioxidant and anti-inflammatory pathways (17). Recent investigations have reported that these breakdown VSCs are responsible for several beneficial health effects (18, 19), with their anticarcinogenic action being the best documented (20), in addition to their well-known contribution to the typical smell and flavor of *Allium* and *Cruciferae* vegetables (18, 21).

Despite the potential impact of VSCs in vegetables, previous studies have focused on the analysis of non-volatile sulfur containing compounds such as glucosinolates based on liquid chromatography system (22–24). In relation to the VSC analysis in food, major VSCs (i.e., dimethyl sulfide, dimethyl trisulfide, methional, hydrogen sulfide, etc.) were identified as potential odorants in cheddar cheese, wine, tropical fruits, and fragrant rice (25–28). There is still a lack of information about the compositions of VSCs in various vegetables and their cooking conditions. VSCs have been quantified in food using headspace solid-phase microextraction (HS-SPME) followed by gas chromatography (GC)-pulsed flame photometric detection (PFPD) (29, 30), GC-chemiluminescence detection (SCD) (28) and GC-mass spectrometry (MS) (26, 31). In VSC analysis, HS-SPME provides many advantages over conventional sample preparation techniques, including simplicity, speed, solvent-free extraction, and minimal sample manipulation (32). The application of MS detection enables both the acquisition of qualitative and quantitative information about the sample and the determination of the molar masses of the analytes (33). Therefore, HS-SPME-GC–MS can be a promising technique for the extraction of volatile compounds in foods, allowing for the rapid and convenient identification and quantification of volatile components without destruction (34, 35).

The aims of this study were to (1) validate the analytical method of VSCs in vegetable matrices using HS-SPME-GC/MS technique, (2) investigate the compositions of VSCs in various vegetables consumed in Korea and (3) characterize the profile of VSCs depending on the type of vegetables and cooking conditions.

## 2 Materials and methods

### 2.1 Chemicals

Volatile standards (dimethyl sulfide, allyl methyl sulfide, diallyl sulfide, dimethyl disulfide, diallyl disulfide, allyl methyl disulfide, allyl propyl disulfide, methyl propyl disulfide, dimethyl trisulfide, diallyl trisulfide, 2,5-dimethyl thiophene, allyl mercaptan, erucin, allyl isothiocyanate, phenethyl isothiocyanate, 3-methylthiopropyl isothiocyanate) were purchased from Sigma-Aldrich Co. (St. Louis, MO, United States). All other reagents were of analytical grade.

### 2.2 Samples

The samples used in this study were obtained from the Rural Development Administration (RDA, Republic of Korea) in 2023 and were promptly stored in a deep freezer until analysis. A total of 58 different vegetables, including various types and their cooked forms, were analyzed. Sample information is as follows: *Cruciferae* vegetables include broccoli [raw, sample code (#) 1; blanched, # 2], broccoli stem (raw, # 3; blanched, # 4), kohlrabi (raw, # 5), Korean shepherd’s purse (*Nangi*) (raw, # 6; blanched, # 7), kale (raw, # 8), Korean spring cabbage (raw, # 9; blanched, # 10), and cabbage (raw, # 11; blanched, # 12; steamed, # 13). *Allium* vegetables include Korean wild chive (raw, # 14), garlic (raw, # 15), garlic clove (raw, # 16), garlic sprout (raw, # 17), garlic root (raw, # 18), and green onion (raw, # 19; grilled, # 20; blanched, # 21). Capsicum species included yellow paprika (raw, # 22; stir-fried, # 23), red paprika (raw, # 24; stir-fried, # 25), orange paprika (raw, # 26; stir-fried, # 27), red bell pepper (raw, # 28; stir-fried, # 29), and green bell pepper (raw, # 30; stir-fried, # 31). Green leafy vegetables include red romaine butterhead lettuce (raw, # 32), green romaine butterhead lettuce (raw, # 33), red skirt lettuce leaf (raw, # 34), tree skirt lettuce leaf (raw, # 35), chicory (raw, # 36), pak choi (raw, # 37; blanched, # 38), and gom-chwi (raw, # 39; blanched, # 40). Mushroom included enoki mushroom (raw, # 41; boiled, # 42; grilled, # 43), king oyster mushroom (raw, # 44; grilled, # 45; boiled, # 46), and shiitake mushroom (dried, # 47; boiled and dried, # 48; boiled, # 49; grilled, # 50; raw, # 51). Others include bracken (*Gosari*) (boiled and dried, # 52; boiled, dried and dried, # 53), burdock (raw, # 54; blanched, # 55; stir-fried, # 56), and curled mallows (raw, # 57; blanched, # 58). The detailed list of samples is indicated in Supplementary Tables S1.

The different cooking condition was shown in Table 1. The most common cooking methods used by the Korean population (i.e., blanching, boiling, steaming, stir-frying, and grilling) were applied (36). The effect of cooking on VSCs of five vegetables (broccoli, Korean shepherd’s purse, green onion, napa cabbage, and cabbage) chosen on the basis of their different morphological feature and VSC compositions. Cooking conditions were optimized by preliminary experiments carried out for each vegetable. For all cooking treatments, the minimum cooking time to reach a similar tenderness for an adequate palatability and taste, according to the Korean eating habits, was used.

**Table 1:** Cooking conditions of various vegetables.

#	Vegetable	Cooking method	Volume of water	Cooking time	Preparation	After cooking
2	Broccoli	Blanching	10-fold the sample	30 s	–	30 min at RT
4	Broccoli stem	Blanching	10-fold the sample	30 s	–	30 min at RT
7	Korean shepherd’s purse	Blanching	10-fold the sample	1 min	–	–
10	Korean spring cabbage	Blanching	10-fold the sample	30 s	–	30 min at RT
12	Cabbage	Blanching	10-fold the sample	1 min	Cut into eighths, blanch, and then remove the core	30 min at RT
13		Steaming	steamer rack is not submerged	10 min	Cut into quarters, steam, and then remove the core	30 min at RT
20	Green onion	Grilling	–	Stem 8 min, Leaf 3 min	–	30 min at RT
21		Blanching	10-fold the sample	50 s	–	30 min at RT
23	Yellow paprika	Stir-frying	–	4 min 30 s	–	30 min at RT
25	Red paprika	Stir-frying	–	4 min 30 s	–	30 min at RT
27	Orange paprika	Stir-frying	–	4 min 30 s	–	30 min at RT
29	Red bell pepper	Stir-frying	–	4 min 30 s	–	30 min at RT
30	Green bell pepper	Stir-frying	–	4 min 30 s	–	30 min at RT
38	Pak choi	Blanching	10-fold the sample	30 s	–	30 min at RT
40	Gom-chwi	Blanching	10-fold the sample	30 s	–	30 min at RT
42	Curled mallows	Blanching	10-fold the sample	3 min	–	30 min at RT
44	Enoki mushroom	Boiling	5 L	1 min 30s	Cut into 0.5 cm thickness, divide into 500 g portions (mix 2 products in a 1:1 ratio)	–
45		Grilling	–	6 min	Cut into 0.5 cm thickness, divide into 500 g portions (mix 2 products in a 1:1 ratio)	–
46	King oyster mushroom	Grilling	–	6 min	Cut into 0.5 cm thickness, divide into 500 g portions (mix 2 products in a 1:1 ratio)	–
47		Boiling	5 L	1 min 30s	Cut into 0.5 cm thickness, divide into 500 g portions (mix 2 products in a 1:1 ratio)	–
49	Shiitake mushroom	Drying	1.4 L	5 min	–	–
50		Boiling and drying	1Soaking; 5 L, Boiling; 1.4 L	Soaking;2 h, boiled; 5 min	Separate the cap and stem	–
51		Boiling	–	2 min 30 s	–	–
52		Grilling	–	6 min	Separate the cap and stem	–
54	Bracken	Boiling	31.1-fold the sample	2 h	–	30 min at RT
55		Drying	10-fold the sample	-	–	–
57	Burdock	Blanching	10-fold the sample	3 min	–	–
58		Stir-frying	–	6 min	–	30 min at RT

*RT, room temperature.

Organic cabbage juices containing 45% cabbage extracts, 5% fructo-oligosaccharide, and 50% distilled water were purchased from local market to validate the analytical method of VSCs in vegetable matrix. The cabbage juices were thoroughly mixed and aliquoted before being stored at −80°C until analysis.

### 2.3 Analysis of VSCs by HS-SMPE/GC–MS

Preliminary experiments were performed to determine the analytical features, including the type and coating of the SPME fiber and the column. Two SPME fibers coated with carboxyl/polydimethylsiloxane (CAR/PDMS) and divinylbenzene (DVB)/CAR/PDMS (50/30 μm, Supelco, Bellefonte, PA, United States) were tested with three capillary columns (30 m × 0.25 mm i.d., 0.25 μm film thickness; Agilent, Santa Clara, CA, United States): HP-FFAP, HP-5MS, and DB-WAX for the analysis of VSCs in cabbage drink. The abundance of the peaks after extraction with the DVB/CAR/PDMS fiber and the DB-WAX column was slightly higher than under other conditions (Supplementary Figure S1). Therefore, DVB/CAR/PDMS fiber and DB-WAS column were chosen for VSC analysis of other vegetables.

The fresh vegetables were ground using mortar and pestle under liquid nitrogen. 2–3 g of ground vegetable samples were placed into a 20 mL headspace vial containing internal standard (ethyl methyl sulfide 4.16 μg/mL) and 3–4 mL of distilled water, and thermostatic autosampler tray at 45°C for 10 min before HS-SPME. Headspace volatiles were captured on SPME fiber coated with DVB/CAR/PDMS for 40 min, and thermally desorbed at 250°C for 10 min in the GC injector port (split ratio; 10:1). GC–MS (Agilent 8890A/5977B MSD Series, Agilent Technologies, Santa, Clara, CA, United States) system was operated with the full scan mode (m/z 35–550). The interface was kept at 230°C and the ionization mode was electron impact (70 eV). The volatiles were separated on a DB-WAX capillary. The GC oven temperature program was as follows: start at 35°C for 2 min, 35–45°C at a rate of 2°C/min, then 45–130°C at a rate of 5°C/min, and finally hold at 225°C at a rate of 10°C/min for 5 min. Helium gas was used as carrier gas with a constant flow rate of 1.5 mL/min. The transfer line temperature was kept at 250°C. Identification of VSCs was carried out by comparison of the volatile sample mass spectra authentic chemicals and NIST library (ver.20). A series of n-alkane (C7-C30) standards was employed to determine the linear retention index (RI) of each compound. The quantification of VSCs was based on the calibration curves. The quantification of VSCs was performed with calibration curve of pure standard. When the authentic standards were not available, tentative identifications were based on the NIST library and a comparison of RI in the literature. The tentative quantification of each compound in the samples was calculated by determining the ratio between the area of the analyte and that of the internal standard.

### 2.4 Method validation

The method validation was conducted in terms of linearity of standard calibration curves, sensitivity (limit of detection, LOD and limit of quantification, LOQ), and precision (inter-day and intra-day). For the standard calibration curves, the different authentic standards with internal standard were added into a 20 mL-vial containing 6 mL of distilled water. The linearity was obtained by calibration curves constructed with peak area ratios of analyte to internal standard against nominal analyte concentration using different analyte concentrations shown in Table 2. Limit of detection (LOD, the analyte concentration for signal/noise = 3) and quantification (LOQ, the analytes concentration for signal/noise = 10) were determined. The precision was evaluated using cabbage drink, which contained the major VSCs such as dimethyl sulfide, dimethyl disulfide, and dimethyl trisulfide, predominantly found in the vegetables used in this study. Cabbage juice (5 mL) was combined with 1 mL of distilled water into a 20 mL headspace vial containing internal standard (ethyl methyl sulfide 4.16 μg/mL). Three replicate analyses were performed on the same day (intra-day assay) and three consecutive days (inter-day assay).

**Table 2:** Validation parameters of HS-SPME/GC–MS method for volatile sulfur compounds.

No.	Compounds	Calibration equations	Linear range (μg/mL)	LOD (μg/mL)^a^	LOQ (μg/mL)^b^	Correlation coefficient (*r*^2^)	Intermediate precision, RSD (%)	Repeatability, RSD (%)
1	Dimethyl sulfide	*y* = 0.0701x-0.054	0.259–16.6	0.028	0.085	0.9911	7.14	9.37
2	Dimethyl disulfide	*y* = 0.627x-0.0508	0.032–1.0375	0.008	0.026	0.9985	4.22	9.45
3	Dimethyl trisulfide	*y* = 2.2394x-0.0038	0.016–2.075	0.008	0.02	0.9975	7.71	8.07

^a^The limits of detection of the method. ^b^The limits of quantitation of the method.

### 2.5 Statistical analysis

All experimental data were analyzed by the one-way analysis of variance (ANOVA) followed by the Tukey’s honestly significant difference (Tukey’s HSD) test using GraphPad 8 (United States). Variables with *p*-value <0.05 (from ANOVA) were considered as potential markers which could discriminate different groups. Data were analyzed using Principal Component Analysis (PCA) applied to the data matrix using the XLSTAT 2018 system software (Addinsoft, United States). A heat map was generated using MetaboAnalyst 6.0.^1^

## 3 Results and discussion

### 3.1 Method validation

The SPME provides several analytical advantages such as simplicity, speed, solvent-free extraction, and minimal sample manipulation, compared to conventional extraction methods like liquid–liquid extraction (LLE), solvent-assisted flavor evaporation (SAFE), and simultaneous distillation extraction (SDE). The elimination of solvents is necessary for LLE, SAFE, and SDE (37). Several studies have used SPME to develop and validate methods for the quantification of VSCs in various food matrices, such as cheese (38) wine (39), and milk (40). Therefore, this study established a comprehensive analysis using HS-SPME-GC–MS for extracting VSCs in different vegetables, enabling the rapid and convenient identification and quantification of VSCs without causing any destruction.

**Figure 1:**
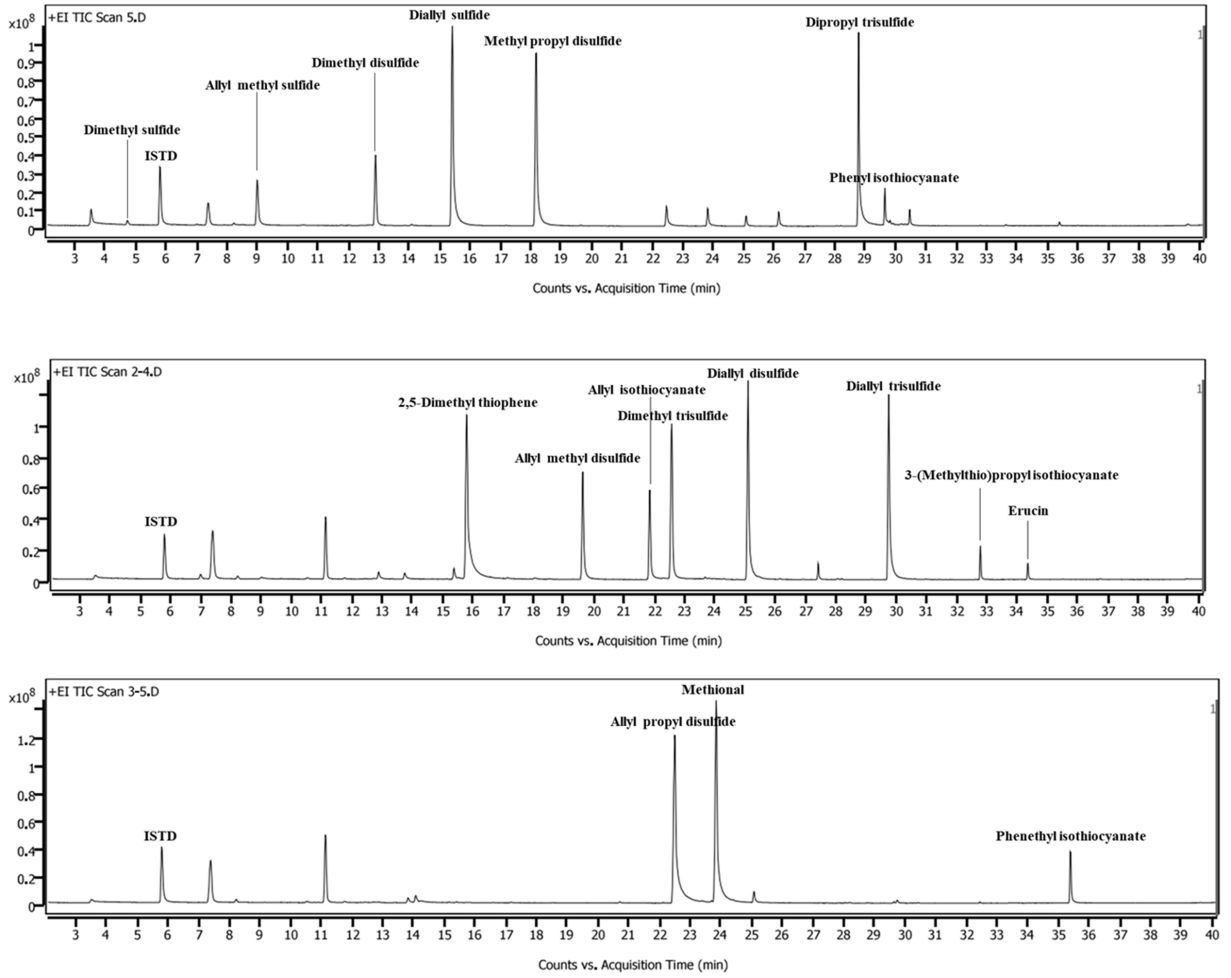
Chromatogram of volatile sulfur compounds.

The HS-SPME/GC–MS method was validated in terms of LOD, LOQ, linearity, and precision for VSC analysis of different vegetables (Table 2). All peaks for VSCs in the chromatograms were completely separated from peaks for other constituents (Figure 1). Dimethyl sulfide, dimethyl disulfide, and dimethyl trisulfide in the cabbage drink were identified by comparing the retention times and mass spectra of the samples with those of the authentic standards (Supplementary Figure S2). The calibration curves for dimethyl sulfide, dimethyl disulfide, and dimethyl trisulfide exhibited excellent linearity with the coefficient of determination (*r*^2^) values higher than 0.99 across the concentration ranges of analytes, indicating a strong linear relationship between concentration and the GC–MS signal. The obtained LODs ranged between 0.008 and 0.028 μg/mL for dimethyl sulfide and dimethyl trisulfide, respectively. The obtained LOQs ranged between 0.02 and 0.085 μg/mL for dimethyl sulfide and dimethyl trisulfide, respectively. The LOD and LOQ were low enough to quantify the VSCs in real samples. The relative standard deviation (RSD) of the analytes were all between 4.22 and 9.45%, which were within the control range and demonstrated the high reproducibility of the method. Thus, the developed method was able to analyze the VSCs that came from different types of vegetables and their cooking conditions. The calibration curves for other VSCs found in various vegetables showed good linearity, with *r*^2^ values higher than 0.95 across the concentration ranges of analytes. The summary of validation results is shown in Table 3.

**Table 3:** Calibration equations, linear range and correlation coefficient of volatile sulfur compounds in HS-SPME/GC–MS method.

No.	Compounds	Calibration equations	Linear range (μg/mL)	Correlation coefficient
1	Allyl isothiocyanate	*y* = 2.133x + 1.516	0.18–35	0.9753
2	Allyl methyl disulfide	*y* = 1.690x + 0.200	0.04–31	0.9886
3	Dimethyl disulfide	*y* = 0.496x + 0.104	0.01–7.98	0.9919
4	Dimethyl sulfide	*y* = 0.070x-0.054	0.004–1.1	0.9911
5	Dimethyl trisulfide	*y* = 1.674x + 0.302	0.003–26.61	0.9936
6	Erucin	*y* = 0.663x + 0.645	0.03–5	0.9495
7	Allyl mercaptan	*y* = 0.151x + 0.137	0.013–27.7	0.9769
8	Diallyl sulfide	*y* = 2.075x + 0.010	0.002–27.7	0.9822
9	Allyl methyl sulfide	*y* = 0.564x-0.057	0.013–27.7	0.9848
10	Allyl propyl disulfide	*y* = 5.333x-0.376	0.003–27.7	0.9927
11	Diallyl disulfide	*y* = 4.951x + 0.096	0.003–27.7	0.9971
12	Diallyl trisulfide	*y* = 0.763x-0.742	0.027–27.7	0.9492
13	Dipropyl disulfide	*y* = 0.358x + 0.062	0.004–0.5186	0.9923
14	Dipropyl trisulfide	*y* = 0.266×0.006	0.004–0.5186	0.9545
15	3-methylthiopropyl isothiocyanate	*y* = 1.814x-0.386	0.064–8.3	0.9971

### 3.2 Compositions of VSCs in different types of vegetables

**Figure 2:**
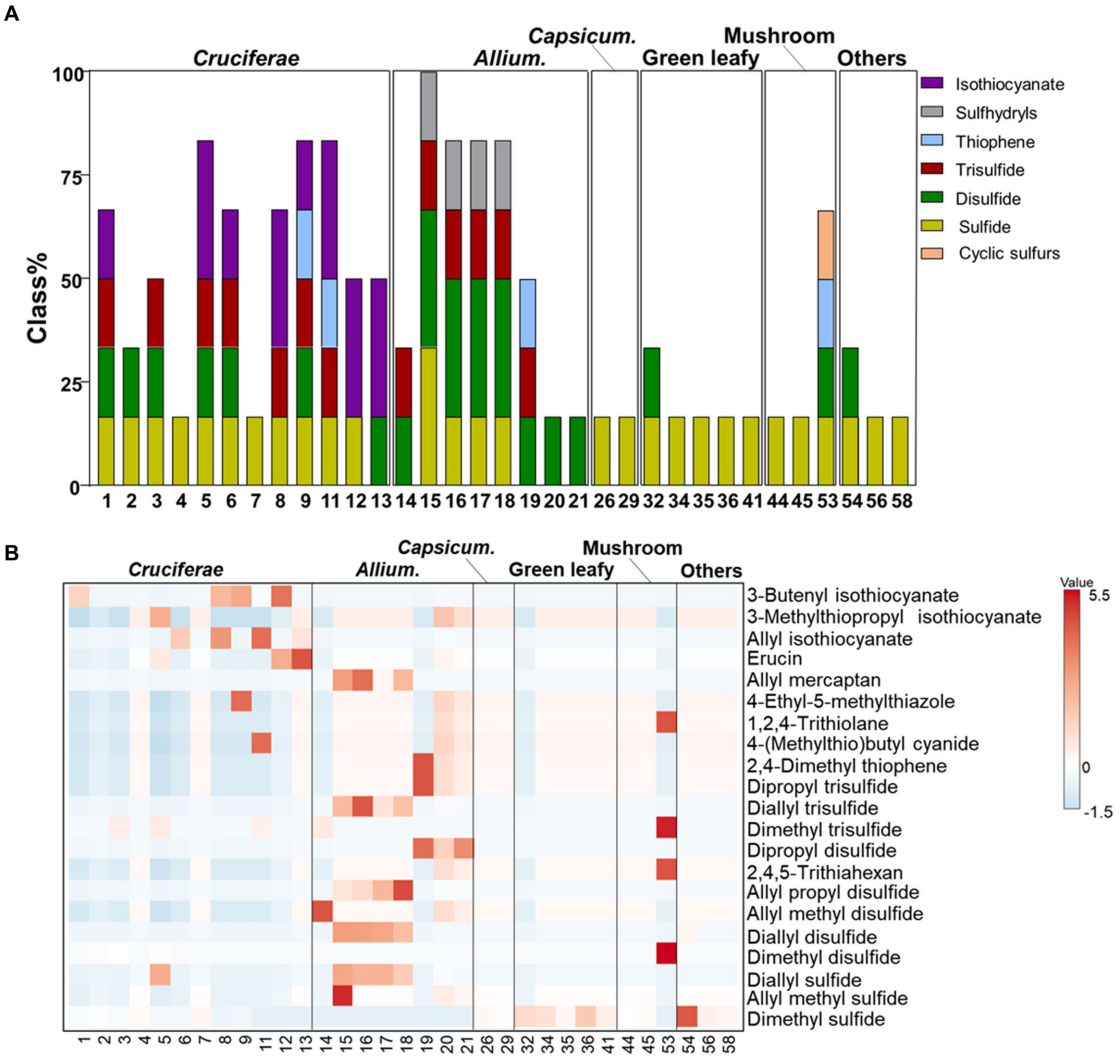
Relative contents **(A)** and heatmap **(B)** of volatile sulfur compounds in different vegetable groups.

The validated HS-SPME/GC–MS method was conducted to analyze the composition of VSCs in different vegetable groups and the cooked vegetables. The result showed that 21 VSCs, including 3 sulfides, 6 disulfides, 3 trisulfides, 3 thiophenes, 4 isothiocyanate, 1 sulfhydryls, and 1 sulfur-containing cyclic compound were identified and quantified in *Allium* species, *Cruciferae*, *Capsicum* species, green leafy vegetables, and mushrooms (Supplementary Table S2 and Figure 2A).

Although 21 VSCs have been reported in various foods, the compositions of VSCs in different vegetables and their cooking conditions were reported exclusively in this study. Particularly, VSCs in *Nangi*, *Bomdong*, *Gosari*, and burdock were analyzed for the first time. Dimethyl sulfide, dimethyl disulfide, dimethyl trisulfide, and allyl isothiocyanate were identified in *Nangi* (#6), whereas allyl isothiocyanate diminished during blanching. *Bomdong* had relatively high levels of isothiocyanates, such as allyl isothiocyanate and 3-butenyl isothiocyanate, which were previously identified in *Brassica juncea* (raya) seeds (41). Dried shiitake mushrooms (#47) contained a diverse range of VSCs, such as dimethyl disulfide, dimethyl trisulfide, 2,4,5-trithiahexane, and 1,2,4-trithiolane. Notably, 2,4,5-trithiahexane and 1,2,4-trithiolane were only found in dried shiitake mushrooms. The content of cyclic sulfur compounds increased during heating, with a significant elevation of 1,2,4-trithiolane after treating shiitake mushrooms at 70°C compared to the fresh ones (42). Dried mushrooms are generally produced using a hot-air drying machine, and the dried shiitake mushrooms used in this study might have been affected by the high-temperature heat treatment of the air-drying process.

*Cruciferae* and *Allium* vegetables had relatively higher amount of VSCs compared to other vegetables. The VSCs of *Cruciferae* vegetables are abundant in sulfides (i.e., dimethyl sulfide, dimethyl disulfide, and dimethyl trisulfide) and isothiocyanates (i.e., allyl isothiocyanate, 3-methylthiopropyl isothiocyanate, erucin, and 3-butenyl isothiocyanate). Raw kohlrabi (#5) had the highest level of VSCs, which were mainly composed of dimethyl sulfide (2.97 ± 0.47 mg/100 g), dimethyl disulfide (7.05 ± 0.07 mg/100 g), dimethyl trisulfide (11.14 ± 1.32 mg/100 g), erucin (1.48 ± 0.35 mg/100 g), and 3-methylthiopropyl isothiocyanate (0.68 ± 0.12 mg/100 g). Raw cabbage (# 11) contained dimethyl sulfide, dimethyl trisulfide, allyl isothiocyanate, 5-methylthiophetanonitrile, and 3-butenyl isothiocyanate. However, erucin was produced by blanching and steaming. *Allium* vegetables contained high levels of allyl groups (i.e., allyl methyl allyl methyl sulfide, diallyl sulfide, allyl propyl disulfide, allyl mercaptan etc.) and 2,4-dimethyl thiophene. Allyl methyl sulfide, which alleviates hyperglycemia-mediated hepatic oxidative stress and inflammation, was found only in raw garlic (#15) (43). Korean wild chive was mainly composed of disulfides such as dimethyl disulfide (0.7 ± 0.20 mg/100 g), allyl methyl disulfide (1.78 ± 0.08 mg/100 g), and dimethyl trisulfide (6.05 ± 0.06 mg/100 g).

The major VSCs of raw garlic samples, including garlic (# 15), garlic clove (# 16), garlic sprout (# 17), and garlic root (# 18), were diallyl sulfide (1.55 ± 0.01 ~ 2.27 ± 0.03 mg/100 g), diallyl disulfide (15.47 ± 1.18 ~ 21.24 ± 1.32 mg/100 g), allyl propyl disulfide (0.50 ± 0.00 ~ 1.98 ± 0.12 mg/100 g), allyl trisulfide (14.56 ± 0.07 ~ 52.67 ± 3.36 mg/100 g), and allyl mercaptan (3.46 ± 0.31 ~ 20.69 ± 6.54 mg/100 g). Diallyl sulfide and diallyl disulfide have therapeutic effects on inflammatory bowel disease, which induce the decrease of IP-10, IL-6, hydrogen sulfide, and nitric oxide, as well as in the expression of STAT-1 observed in intestinal cells stimulated with IFN-γ (44). Raw green onion (#19) contained only dipropyl disulfide, dipropyl trisulfide, and 2,4-dimethyl thiophene, which are partially responsible for its bioactivity (45). A previous study also reported that the major volatile in onion headspace has been reported to be dipropyl disulfide, followed by other mono- and disulfides, thiols, and thiophenes (46). Erucin, a promising anti-carcinogenic agent identified in rocket salad and arugula (*Eruca sativa*), was found only in raw kohlrabi (#5), blanched cabbage (#12), and steamed cabbage (#13) in this study (47, 48).

Even though ultra-volatile and highly reactive compounds such as hydrogen sulfide (H2S), methyl mercaptan (MeSH), and ethyl mercaptan (EtSH) can be found in Brassica and *Allium* species (49, 50) the methods employed in our study were not designed for their quantification. The concentrations of these compounds can vary significantly based on variety and growing conditions, and they may be present in very low quantities. Our study focused on profiling dimethyl sulfides, isothiocyanates, and allyl compounds.

Collectively, the present study demonstrated that the levels of dietary VSCs vary depending on the type of vegetable and the cooking conditions. Notably, isocyanates were abundant in *Cruciferae*, while allyl groups and propyl sulfides were prevalent in garlic and green onion, respectively. Most cooked vegetables contained lower levels of VSCs compared to their fresh counterparts, except for erucin in cooked cabbage. The diverse compositions of VSCs may not only contribute to the distinct flavors of each vegetable but also influence their different health benefits.

### 3.3 Profile of VSCs in different vegetable groups

Heatmap analysis was applied to illustrate the different compositions of VSCs across the various vegetable groups (Figure 2B). Most vegetables contained sulfide derivatives. *Cruciferae* and *Allium* vegetables had higher concentrations of VSCs, while *Capsicum* species, green leafy vegetables, and mushrooms had comparatively lower VSC content. *Cruciferae* and *Allium* vegetables had relatively higher amounts of VSCs compared to other vegetables. The largest group of *Cruciferae* -released VSCs was isothiocyanates, mainly composed of allyl isothiocyanate, 3-methylthiopropyl isothiocyanate, and 3-butenyl isothiocyanate. Erucin was found only in cooked cabbages. Among the VSCs, allyl groups (i.e., allyl methyl sulfide, diallyl sulfide, allyl propyl disulfide, allyl mercaptan, etc.) and 2,4-dimethyl thiophene were significantly abundant in *Allium* vegetables. The distinct VSC patterns in *Cruciferae* and *Allium* vegetables are responsible for their strong flavors and aromas, which are often mellowed by cooking processes (51). Raw Shiitake mushrooms have relatively higher amounts of disulfides, such as dimethyl disulfide, dimethyl sulfide, 2,4,5-trithiahexane, and cyclic sulfurs (i.e., 1,2,4-trithiolane) compared to other mushrooms, as reported in previous studies. The major volatile sulfur compounds, 2,4,5-trithiahexane and 1,2,4-trithiolane, significantly contribute to the complex aroma of Shiitake mushrooms, which varies depending on the cooking conditions (52). Green leafy vegetables, bracken, and burdock are more abundant in dimethyl disulfide, a metabolite produced during various biological processes in plants (53).

**Figure 3:**
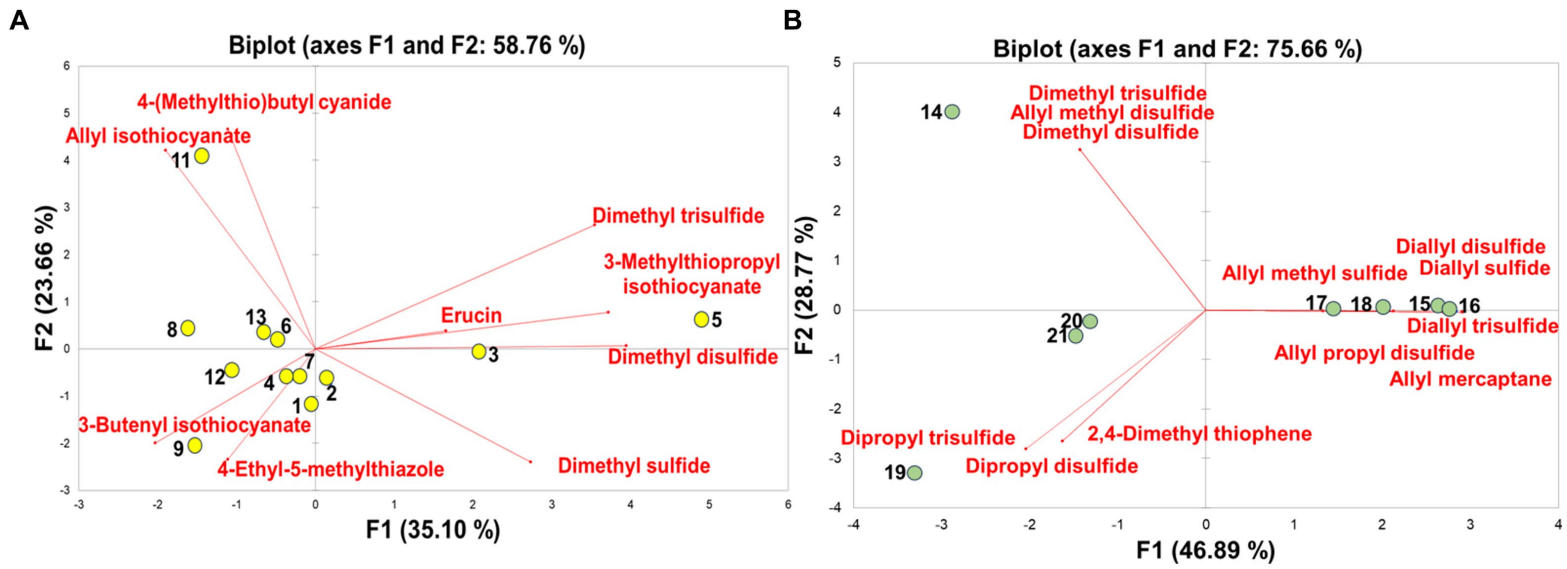
Principal component analysis of volatile sulfur compounds in *Cruciferae* **(A)** and *Allium* **(B)** vegetables.

The PCA was performed using the VSC profiling data of different vegetables to reveal the sample clustering pattern, provide an overview of the trends, and determine putative outliers. As depicted in Supplementary Figure S3, the PCA score plot revealed that differences between vegetables were generally unclear, except for one Shiitake mushroom sample and several *Allium* vegetable samples. The first two principal components (PCs) accounted for 42% of the total variance [PC1 (*x*-axis) = 23.42% and PC2 (*y*-axis) = 18.53%]. In the absence of distinctive VSCs among the vegetables, most samples contained sulfides such as dimethyl sulfide and dimethyl disulfide, resulting in ambiguous VSC patterns and complicating the classification process. Therefore, PCA was separately conducted for *Cruciferae* and *Allium* vegetables, which not only exhibited distinctive VSCs but also had relatively higher amounts of these compounds. Figure 3A showed distinct clustering patterns among the *Cruciferae* vegetables. The first two PCs accounted for 58.76% of the total variance (PC1 = 35.10% and PC2 = 23.66%). Broccoli stem (# 3), Kohlrabi (# 5), and blenched broccoli (# 2) were positioned on the positive side of the PC 1 axis, while the other vegetables were on the negative side. Among the samples positively placed on the PC 1, kohlrabi was significantly associated with erucin, 3-methylthiopropyl isothiocyanate, dimethyl disulfide and dimethyl trisulfide. Korean spring cabbage (# 9) was negatively placed on both PC 1 and PC 2 and was significantly related to 4-ethyl-5-methylthiazole and 3-butenyl isothiocyanate. Cabbage (# 11) was located on the positive value of PC 1 axis and the negative value of PC 2 axis, which was linked to allyl isothiocyanate. The potential role of *Cruciferae* vegetables in cancer chemoprevention is attributed to the bioactivity of their glucosinolate hydrolysis products, namely isothiocyanates, which are suggested to protect against common cancer types such as lung, prostate, breast, and colon cancers (54).

As shown in Figure 3B, the first two components of the PCA accounted for 75.66% of the total VSCs within samples: PC1 and PC2 explained 46.89 and 28.77% of the total variation, respectively. Garlics (# 15, 16, 17, and 18) were located on the positive values of the PC 1 axis, whereas the other vegetables were on the negative values. Garlics were significantly associated with allyl groups such as diallyl sulfide, diallyl disulfide, diallyl trisulfide, allyl methyl sulfide, allyl propyl disulfide, and allyl mercaptan. In garlics, these allyl compounds, which contribute to the pungent flavor and odor of garlic, are decomposed from allicin (55). The antioxidant activities of garlics can be attributed to these allyl compounds (56) (ref.), which also influence therapeutic effects, including anti-inflammatory, anti-obesity, and cancer prevention potentials (57, 58). Green onions (# 19, 20, and 21) were negatively placed on both PC 1 and PC 2 and was significantly associated with 2,4-dimethyl thiophene, dipropyl trisulfide, and dipropyl disulfide. Korean wild chive (# 14) was located on the positive value of PC 1 axis and the negative value of PC 2 axis, which was related to dimethyl disulfide, dimethyl trisulfide, and allyl methyl disulfide.

These findings highlight the unique VSC compositions in specific vegetables, offering valuable insights into their distinct biochemical profiles and potential health benefits depending on the type of vegetable.

### 3.4 Changes in VSCs in vegetables depending on different cooking conditions

Vegetables are often consumed raw, but they also frequently undergo heat treatment in commercial processing plants and household kitchens before consumption (59). Cooking vegetables can alter the composition of VSCs, thereby influencing their nutritional quality.

**Figure 4:**
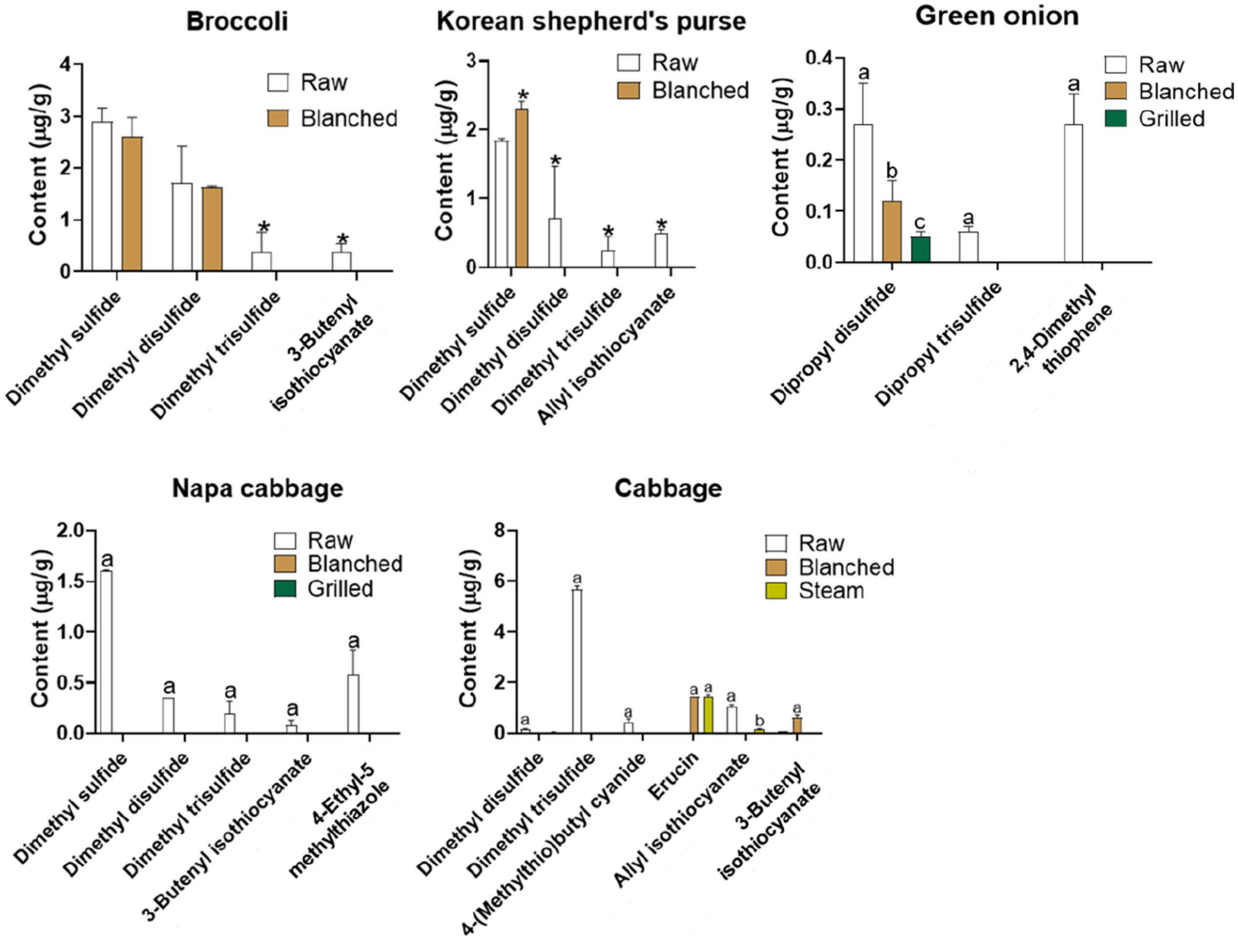
Profile of volatile sulfur compounds in vegetables according to different cooking methods. Data were compared using Tukey HSD or *T*-test. In broccoli and Korean shepherd’s purse, *p < 0.05 represent significant difference of raw and blanched group. In green onion, napa cabbage and cabbage, different letters indicate a significant difference between groups at the *p* < 0.05.

The present examined how cooking affects the VSCs in different vegetables. Figure 4 illustrates the differences in VSC compositions in broccoli, shepherd’s purse, cabbage, and napa cabbage under various cooking conditions. Most VSCs decreased after blanching, steaming, and grilling, except for dimethyl sulfide in Korean shepherd’s purse and erucin in cabbage. Previous studies have demonstrated that the volatile compounds in vegetables are affected by thermal degradation (60). Raw *Allium* vegetables contain higher amounts of disulfides, mainly diallyl disulfide and allyl (E)-1-propenyl disulfide, compared to cooked samples (61, 62). In this study, blanched broccoli showed reduced contents of dimethyl trisulfide and 3-butenyl isothiocyanate, although dimethyl sulfide and dimethyl disulfide remained stable during cooking. In Korean shepherd’s purse, the content of dimethyl sulfide was higher in the blanched sample than in the raw sample, while dimethyl disulfide, dimethyl trisulfide, and allyl isothiocyanate were destroyed after blanching. The contents of dipropyl disulfide, dipropyl trisulfide, and 2,4-dimethyl thiophene were significantly reduced in blanched and grilled green onions. Cooked napa cabbage showed a decrease in all VSCs detected in the raw sample. Major VSCs in napa cabbage were not detected after cooking. Changes in VSCs in cabbage were diverse after cooking. In particular, dimethyl disulfide, dimethyl trisulfide, and 4-(methylthio)butyl cyanide were destroyed after blanching and steaming. However, the content of erucin was enhanced in blanched and steamed cabbage, compared to raw cabbage. Blanching also caused an increase in the 3-butenyl isothiocyanate content. In the cooked cabbage, isothiocyanate such as erucin and 3-butenyl isothiocyanate might be generated from the thermal decomposition of glucosinolates such as glucoraphanin. Even though isothiocyanates in *Cruciferae* vegetables are typically produced through the primary hydrolytic pathway of glucosinolates catalyzed by myrosinase, the high temperatures and extended processing times used in cooking can inactivate this enzyme (63). These founding suggest that most of cooked vegetables were lower contents of VSCs than that of their fresh counterparts, but it depends on the type of vegetables and compounds.

In conclusion, this study investigated the profile of VSCs in different vegetable groups and the impact of various cooking methods using HS-SPME/GC–MS combined with multivariate chemometric analysis. The analytical method of VSCs was validated with vegetable matrices using HS-SPME-GC/MS technique. The primary objective of this study was to profile the overall volatile sulfur compounds (VSCs) present in different vegetable matrices. Within this scope, the method provided valuable preliminary insights. Future research should focus on employing labeled isotopic standards for the absolute quantification of major VSCs to enhance accuracy and reliability. This approach will address the limitations of the current method and provide more definitive data on the concentration of VSCs in vegetable samples. The compositions of VSCs varied across a range of vegetables, including *Allium* species, *Cruciferae* vegetables, *Capsicum* species, green leafy vegetables, and mushrooms. Notably, isothiocyanate and allyl groups were abundant in *Cruciferae* and *Allium* vegetables, respectively. In cooked vegetables, a reduction in dimethyl sulfide and dimethyl trisulfide was observed, suggesting that heat treatment can lead to the volatilization and reduction of these compounds. Several isothiocyanates in cabbage, such as erucin and 3-butenyl isothiocyanate, increased with cooking, likely due to the thermal decomposition of glucosinolates. The cooking method plays a critical role in modulating the presence and concentration of VSCs in vegetables, with implications for potential health benefits. Future dietary recommendations and culinary practices should consider the influence of cooking methods on the nutritional quality of vegetables to optimize the intake of beneficial sulfur-containing compounds.

## Data availability statement

The datasets presented in this article are not readily available because the datasets used and/or analyzed during the current study are available from the corresponding author on reasonable request. Requests to access the datasets should be directed to jeehye@anu.ac.kr.

## Author contributions

SP: Formal analysis, Investigation, Methodology, Writing – original draft, Software, Validation, Visualization, Writing – review & editing. H-WK: Funding acquisition, Project administration, Writing – original draft. CL: Investigation, Project administration, Writing – original draft. YK: Formal analysis, Investigation, Methodology, Supervision, Validation, Writing – original draft. JS: Conceptualization, Data curation, Formal analysis, Investigation, Methodology, Project administration, Supervision, Writing – original draft.

## Footnotes

^1^ www.metaboanalyst.ca

## References

[1] Schutte L Teranishi R. Precursors of sulfur-containing flavor compounds. Crit Rev Food Sci Nutr. (1974) 4:457–505. doi: 10.1080/10408397409527166

[2] Dordevic D Capikova J Dordevic S Tremlová B Gajdács M Kushkevych I. Sulfur content in foods and beverages and its role in human and animal metabolism: a scoping review of recent studies. Heliyon. (2023) 9:e15452. doi: 10.1016/j.heliyon.2023.e15452 37123936 PMC10130226

[3] Miękus N Marszałek K Podlacha M Iqbal A Puchalski C Świergiel AH. Health benefits of plant-derived sulfur compounds, glucosinolates, and organosulfur compounds. Molecules. (2020) 25:3804. doi: 10.3390/molecules25173804, PMID: 32825600 PMC7503525

[4] Doleman JF Grisar K van Liedekerke L Saha S Roe M Tapp HS. The contribution of alliaceous and cruciferous vegetables to dietary Sulphur intake. Food Chem. (2017) 234:38–45. doi: 10.1016/j.foodchem.2017.04.098, PMID: 28551250 PMC5460521

[5] Landaud S Helinck S Bonnarme P. Formation of volatile sulfur compounds and metabolism of methionine and other sulfur compounds in fermented food. Appl Microbiol Biotechnol. (2008) 77:1191–205. doi: 10.1007/s00253-007-1288-y 18064452

[6] Chu C-C Wu W-S Shieh J-P Chu H-L Lee C-P Duh P-D. The anti-inflammatory and vasodilating effects of three selected dietary organic sulfur compounds from Allium species. J Funct Biomater. (2017) 8:5. doi: 10.3390/jfb8010005, PMID: 28134777 PMC5371878

[7] Zenkov N Menshchikova E Kandalintseva N Oleynik A Prosenko A Gusachenko O. Antioxidant and anti-inflammatory activity of new water-soluble sulfur-containing phenolic compounds. Biochem Mosc. (2007) 72:644–51. doi: 10.1134/S0006297907060077, PMID: 17630909

[8] Cerella C Dicato M Jacob C Diederich M. Chemical properties and mechanisms determining the anti-cancer action of garlic-derived organic sulfur compounds. Anticancer Agents Med Chem. (2011) 11:267–71. doi: 10.2174/187152011795347522 21269260

[9] Yeh Y-Y Liu L. Cholesterol-lowering effect of garlic extracts and organosulfur compounds: human and animal studies. J Nutr. (2001) 131:989S–93S. doi: 10.1093/jn/131.3.989S 11238803

[10] Jung E-S Park S-H Choi E-K Ryu B-H Park B-H Kim D-S. Reduction of blood lipid parameters by a 12-wk supplementation of aged black garlic: a randomized controlled trial. Nutrition. (2014) 30:1034–9. doi: 10.1016/j.nut.2014.02.014, PMID: 24976429

[11] Eilat-Adar S Sinai T Yosefy C Henkin Y. Nutritional recommendations for cardiovascular disease prevention. Nutrients. (2013) 5:3646–83. doi: 10.3390/nu5093646, PMID: 24067391 PMC3798927

[12] Yoo M Lee S Lee S Seog H Shin D. Validation of high performance liquid chromatography methods for determination of bioactive sulfur compounds in garlic bulbs. Food Sci Biotechnol. (2010) 19:1619–26. doi: 10.1007/s10068-010-0229-1

[13] Bhattacharjee S. Dietary phytochemicals in the prevention and therapy of Cancer: modulation of molecular targets In: Rediscovering cancer: from mechanism to therapy. Eds. Sayali Mukherjee, Somali Sanyal, Sonia Chadha New Jersey and Canada: Apple Academic Press (2018). 337–92.

[14] Favela-González KM Hernández-Almanza AY De la Fuente-Salcido NM. The value of bioactive compounds of cruciferous vegetables (Brassica) as antimicrobials and antioxidants: A review. J Food Biochem. (2020) 44:e13414. doi: 10.1111/jfbc.13414 32743821

[15] Narbad A Rossiter JT. Gut glucosinolate metabolism and isothiocyanate production. Mol Nutr Food Res. (2018) 62:e1700991. doi: 10.1002/mnfr.201700991, PMID: 29806736 PMC6767122

[16] Tian S Liu X Lei P Zhang X Shan Y. Microbiota: a mediator to transform glucosinolate precursors in cruciferous vegetables to the active isothiocyanates. J Sci Food Agric. (2018) 98:1255–60. doi: 10.1002/jsfa.8654 28869285

[17] Masella R Mazza G. Glutathione and sulfur amino acids in human health and disease. John Wiley & Sons, Inc. (2009).

[18] Tapiero H Townsend DM Tew KD. Organosulfur compounds from Alliaceae in the prevention of human pathologies. Biomed Pharmacother. (2004) 58:183–93. doi: 10.1016/j.biopha.2004.01.004, PMID: 15164729 PMC6361170

[19] Corzo-Martínez M Corzo N Villamiel M. Biological properties of onions and garlic. Trends Food Sci Technol. (2007) 18:609–25. doi: 10.1016/j.tifs.2007.07.011

[20] Cerella C Kelkel M Viry E Dicato M Jacob C Diederich M. Naturally occurring organic sulfur compounds: an example of a multitasking class of phytochemicals in anti-Cancer research. Anticancer Res Phytochem Bioact Impact Health. (2011) 2:1–40. doi: 10.5772/26003

[21] Js G Breuning S Stahl T Mersch-Sundermann V Mühling KH. Isothiocyanate concentration in kohlrabi (*Brassica oleracea* L. var. gongylodes) plants as influenced by sulfur and nitrogen supply. J Agric Food Chem. (2008) 56:8334–42. doi: 10.1021/jf800399x 18715015

[22] Arora R Arora S Vig AP. Development of validated high-temperature reverse-phase UHPLC-PDA analytical method for simultaneous analysis of five natural isothiocyanates in cruciferous vegetables. Food Chem. (2018) 239:1085–9. doi: 10.1016/j.foodchem.2017.07.059 28873525

[23] Shafaei A Hill CR Hodgson JM Blekkenhorst LC Boyce MC. Simultaneous extraction and quantitative analysis of S-methyl-l-cysteine sulfoxide, sulforaphane and glucosinolates in cruciferous vegetables by liquid chromatography mass spectrometry. Food Chem X. (2024) 21:101065. doi: 10.1016/j.fochx.2023.101065 38187949 PMC10767375

[24] Zhu Q Kakino K Nogami C Ohnuki K Shimizu K. An LC-MS/MS-SRM method for simultaneous quantification of four representative organosulfur compounds in garlic products. Food Anal Methods. (2016) 9:3378–84. doi: 10.1007/s12161-016-0535-1

[25] Mahattanatawee K Rouseff RL. Comparison of aroma active and sulfur volatiles in three fragrant rice cultivars using GC–Olfactometry and GC–PFPD. Food Chem. (2014) 154:1–6. doi: 10.1016/j.foodchem.2013.12.105, PMID: 24518308

[26] Rouseff RL Onagbola EO Smoot JM Stelinski LL. Sulfur volatiles in guava (*Psidium guajava* L.) leaves: possible defense mechanism. J Agric Food Chem. (2008) 56:8905–10. doi: 10.1021/jf801735v, PMID: 18778077

[27] Burbank HM Qian MC. Volatile sulfur compounds in Cheddar cheese determined by headspace solid-phase microextraction and gas chromatograph-pulsed flame photometric detection. J Chromatogr A. (2005) 1066:149–57. doi: 10.1016/j.chroma.2005.01.027, PMID: 15794566

[28] Siebert TE Solomon MR Pollnitz AP Jeffery DW. Selective determination of volatile sulfur compounds in wine by gas chromatography with sulfur chemiluminescence detection. J Agric Food Chem. (2010) 58:9454–62. doi: 10.1021/jf102008r, PMID: 20707415

[29] Fang Y Qian MC. Sensitive quantification of sulfur compounds in wine by headspace solid-phase microextraction technique. J Chromatogr A. (2005) 1080:177–85. doi: 10.1016/j.chroma.2005.05.024, PMID: 16008056

[30] López R Lapena AC Cacho J Ferreira V. Quantitative determination of wine highly volatile sulfur compounds by using automated headspace solid-phase microextraction and gas chromatography-pulsed flame photometric detection: critical study and optimization of a new procedure. J Chromatogr A. (2007) 1143:8–15. doi: 10.1016/j.chroma.2006.12.053, PMID: 17207804

[31] Segurel MA Razungles AJ Riou C Salles M Baumes RL. Contribution of dimethyl sulfide to the aroma of Syrah and Grenache noir wines and estimation of its potential in grapes of these varieties. J Agric Food Chem. (2004) 52:7084–93. doi: 10.1021/jf049160a, PMID: 15537322

[32] Jeleń HH Majcher M Dziadas M. Microextraction techniques in the analysis of food flavor compounds: A review. Anal Chim Acta. (2012) 738:13–26. doi: 10.1016/j.aca.2012.06.006, PMID: 22790695

[33] Hopfgartner G Varesio E. Application of mass spectrometry for quantitative and qualitative analysis in life sciences. Chimia. (2005) 59:321. doi: 10.2533/000942905777676443

[34] Chapman DM Thorngate JH Matthews MA Guinard J-X Ebeler SE. Yield effects on 2-methoxy-3-isobutylpyrazine concentration in cabernet sauvignon using a solid phase microextraction gas chromatography/mass spectrometry method. J Agric Food Chem. (2004) 52:5431–5. doi: 10.1021/jf0400617, PMID: 15315381

[35] Wang H Zhang J Zhu Y Wang X Shi W. Volatile components present in different parts of grass carp. J Food Biochem. (2018) 42:e12668. doi: 10.1111/jfbc.12668

[36] Hwang E-S. Effect of cooking method on antioxidant compound contents in cauliflower. Prev Nutr Food Sci. (2019) 24:210–6. doi: 10.3746/pnf.2019.24.2.210, PMID: 31328127 PMC6615361

[37] Burin VM Marchand S de Revel G Bordignon-Luiz MT. Development and validation of method for heterocyclic compounds in wine: optimization of HS-SPME conditions applying a response surface methodology. Talanta. (2013) 117:87–93. doi: 10.1016/j.talanta.2013.08.037 24209315

[38] Lecanu L Ducruet V Jouquand C Gratadoux JJ Feigenbaum A. Optimization of headspace solid-phase microextraction (SPME) for the odor analysis of surface-ripened cheese. J Agric Food Chem. (2002) 50:3810–7. doi: 10.1021/jf0117107, PMID: 12059164

[39] Slaghenaufi D Tonidandel L Moser S Román Villegas T Larcher R. Rapid analysis of 27 volatile sulfur compounds in wine by headspace solid-phase microextraction gas chromatography tandem mass spectrometry. Food Anal Methods. (2017) 10:3706–15. doi: 10.1007/s12161-017-0930-2

[40] Vazquez-Landaverde PA Torres JA Qian MC. Quantification of trace volatile sulfur compounds in milk by solid-phase microextraction and gas chromatography–pulsed flame photometric detection. J Dairy Sci. (2006) 89:2919–27. doi: 10.3168/jds.S0022-0302(06)72564-4, PMID: 16840607

[41] Bassan P Bhushan S Kaur T Arora R Arora S Vig AP. Extraction, profiling and bioactivity analysis of volatile glucosinolates present in oil extract of *Brassica juncea* var. raya. Physiol Mol Biol Plants. (2018) 24:399–409. doi: 10.1007/s12298-018-0509-4, PMID: 29692548 PMC5911257

[42] Wu C-M Wang Z. Volatile compounds in fresh and processed shiitake mushrooms (*Lentinus edodes* sing.). Food Sci Technol Res. (2000) 6:166–70.

[43] Sujithra K Srinivasan S Indumathi D Vinothkumar V. Allyl methyl sulfide, an organosulfur compound alleviates hyperglycemia mediated hepatic oxidative stress and inflammation in streptozotocin-induced experimental rats. Biomed Pharmacother. (2018) 107:292–302. doi: 10.1016/j.biopha.2018.07.162, PMID: 30098547

[44] Fasolino I Izzo AA Clavel T Romano B Haller D Borrelli F. Orally administered allyl sulfides from garlic ameliorate murine colitis. Mol Nutr Food Res. (2015) 59:434–42. doi: 10.1002/mnfr.201400347, PMID: 25488545

[45] Islam MS Choi H Loots DT. Effects of dietary onion (*Allium cepa* L.) in a high-fat diet streptozotocin-induced diabetes rodent model. Ann Nutr Metab. (2008) 53:6–12. doi: 10.1159/000152868, PMID: 18772584

[46] Kallio H Salorinne L. Comparison of onion varieties by headspace gas chromatography-mass spectrometry. J Agric Food Chem. (1990) 38:1560–4. doi: 10.1021/jf00097a029

[47] Melchini A Costa C Traka M Miceli N Mithen R de Pasquale R. Erucin, a new promising cancer chemopreventive agent from rocket salads, shows anti-proliferative activity on human lung carcinoma A549 cells. Food Chem Toxicol. (2009) 47:1430–6. doi: 10.1016/j.fct.2009.03.024, PMID: 19328833

[48] Azarenko O Jordan MA Wilson L. Erucin, the major isothiocyanate in arugula (*Eruca sativa*), inhibits proliferation of MCF7 tumor cells by suppressing microtubule dynamics. PLoS One. (2014) 9:e100599. doi: 10.1371/journal.pone.0100599, PMID: 24950293 PMC4065051

[49] Chin HW Lindsay R. Volatile sulfur compounds formed in disrupted tissues of different cabbage cultivars. J Food Sci. (1993) 58:835–9. doi: 10.1111/j.1365-2621.1993.tb09370.x

[50] Akpolat H Barringer SA. The effect of pH and temperature on cabbage volatiles during storage. J Food Sci. (2015) 80:S1878–84. doi: 10.1111/1750-3841.12939, PMID: 26121908

[51] Bi J Li B Chen Z Yang Z Ping C Gao Y. Comparative study of volatile flavor compounds in green onion (*Allium fistulosum* L.) processed with different cooking methods. International journal of gastronomy and food. Science. (2024) 35:100878. doi: 10.1016/j.ijgfs.2024.100878

[52] Liu Q Bau T Jin R Cui X Zhang Y Kong W. Comparison of different drying techniques for shiitake mushroom (*Lentinus edodes*): changes in volatile compounds, taste properties, and texture qualities. Lwt. (2022) 164:113651. doi: 10.1016/j.lwt.2022.113651

[53] Ajwa H Ntow WJ Qin R Gao S. Properties of soil fumigants and their fate in the environment In: Hayes' handbook of pesticide toxicology. Netherlands: Elsevier (2010). 315–30.

[54] Melchini A Traka MH. Biological profile of erucin: a new promising anticancer agent from cruciferous vegetables. Toxins. (2010) 2:593–612. doi: 10.3390/toxins2040593, PMID: 22069601 PMC3153205

[55] Okoro BC Dokunmu TM Okafor E Sokoya IA Israel EN Olusegun DO. The ethnobotanical, bioactive compounds, pharmacological activities and toxicological evaluation of garlic (*Allium sativum*): a review. Pharmacol Res Mod Chin Med. (2023) 8:100273. doi: 10.1016/j.prmcm.2023.100273

[56] El-Saber Batiha G Magdy Beshbishy A Wasef LG Elewa YHA Al-Sagan AA Abd el-Hack ME. Chemical constituents and pharmacological activities of garlic (*Allium sativum* L.): a review. Nutrients. (2020) 12:872. doi: 10.3390/nu12030872, PMID: 32213941 PMC7146530

[57] Shang A Cao S-Y Xu X-Y Gan R-Y Tang G-Y Corke H. Bioactive compounds and biological functions of garlic (*Allium sativum* L.). Food Secur. (2019) 8:246. doi: 10.3390/foods8070246 PMC6678835 31284512

[58] Lidiková J Čeryová N Tóth T Musilová J Vollmannová A Mammadova K. Garlic (*Allium sativum* L.): characterization of bioactive compounds and related health benefits In: Herbs and spices-new advances. United Kingdom: IntechOpen (2022).

[59] Kim S Lee S Shin D Yoo M. Change in organosulfur compounds in onion (*Allium cepa* L.) during heat treatment. Food Sci Biotechnol. (2016) 25:115–9. doi: 10.1007/s10068-016-0017-7, PMID: 30263245 PMC6049342

[60] Li H Guan H Zhang X Xing S Liu W Kim I-C. The impact of different cooking methods on the flavor profile of fermented Chinese spicy cabbage. Molecules. (2023) 28:6539. doi: 10.3390/molecules28186539, PMID: 37764317 PMC10535354

[61] Sun J Sun B Ren F Chen H Zhang N Zhang Y. Influence of different frying processes on the flavor characteristics and sensory profile of garlic oil. Molecules. (2019) 24:4456. doi: 10.3390/molecules24244456, PMID: 31817376 PMC6943420

[62] Condurso C Cincotta F Tripodi G Merlino M Verzera A. Influence of drying technologies on the aroma of Sicilian red garlic. Lwt. (2019) 104:180–5. doi: 10.1016/j.lwt.2019.01.026

[63] Angelino D Dosz EB Sun J Hoeflinger JL Van Tassell ML Chen P. Myrosinase-dependent and–independent formation and control of isothiocyanate products of glucosinolate hydrolysis. Front Plant Sci. (2015) 6:831. doi: 10.3389/fpls.2015.00831 26500669 PMC4593958

