# Supplementary material for: Profiles of volatile sulfur compounds in various vegetables consumed in Korea using HS-SPME-GC/MS technique
Source: Front Nutr. 2024 Jul 22;11:1409008. doi: 10.3389/fnut.2024.1409008 (PMC11298481; doi:10.3389/fnut.2024.1409008)
Supplement: Supplementary file 2 [file Image_1.pdf]

Supplementary Material

Profiles of Volatile Sulfur Compounds in Various Vegetables Consumed in Korea using HS-SPME-GC/MS technique

Samuel Park[1], Heonwoong Kim[2], Changjoo Lee[3], Younghwa Kim[4]*, Jeehye Sung[1]*

*** Correspondence:**

Corresponding Author

Younghwa Kim, Ph.D., Professor

Department of Food Science and Biotechnology

Kyungsung University

Busan, 48434, South Korea Tel: +82-51-663-4652 E-mail: younghwakim@ks.ac.kr

Jeehye Sung, Ph.D., Professor

Department of Food Science and Biotechnology

Andong National University

Andong, 36729, South Korea Tel: +82-54-820-5752 E-mail: jeehye@anu.ac.krCorresponding Author: email@uni.edu

1 Supplementary Figures and Tables

1.1 Supplementary Figures

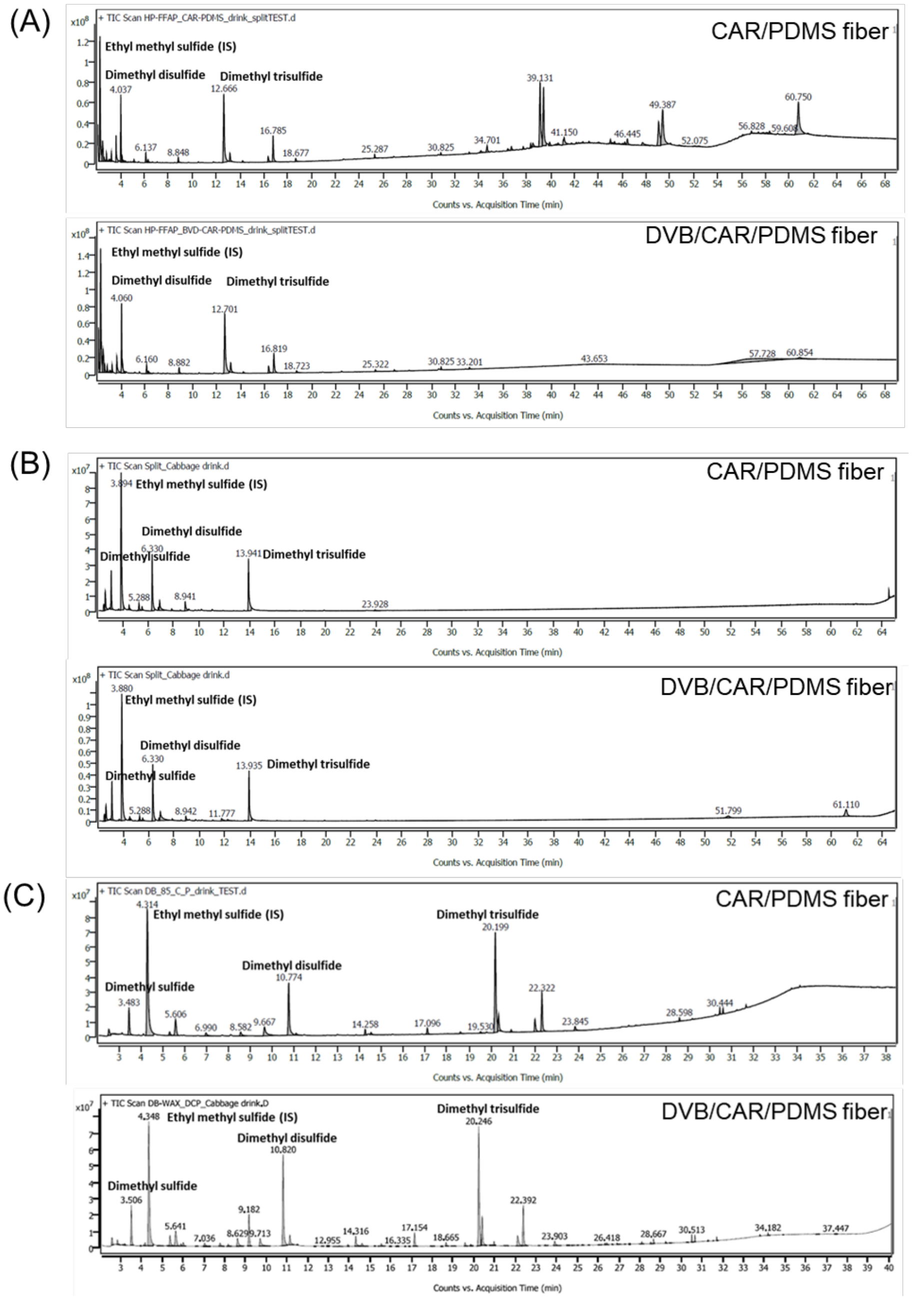

Supplementary Figure 1. Chromatogram of cabbage drink using different types of coatings on the SPME fiber and different columns ((A) HP-FFAP, (B) HP-5MS, and (C) DB-WAX).

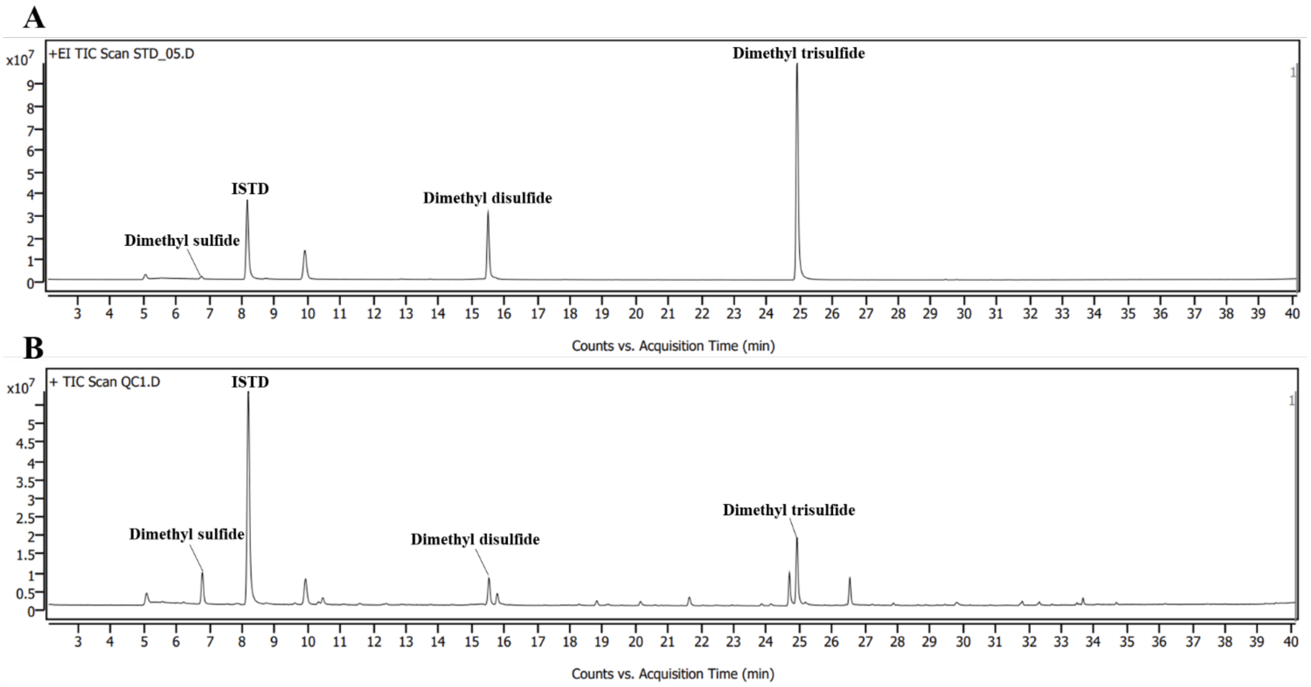

Supplementary Figure 2. Standard chromatogram of standard mixture for HS-SPME/GC-MS method validation. Standard mixture (A) and cabbage drink (B).

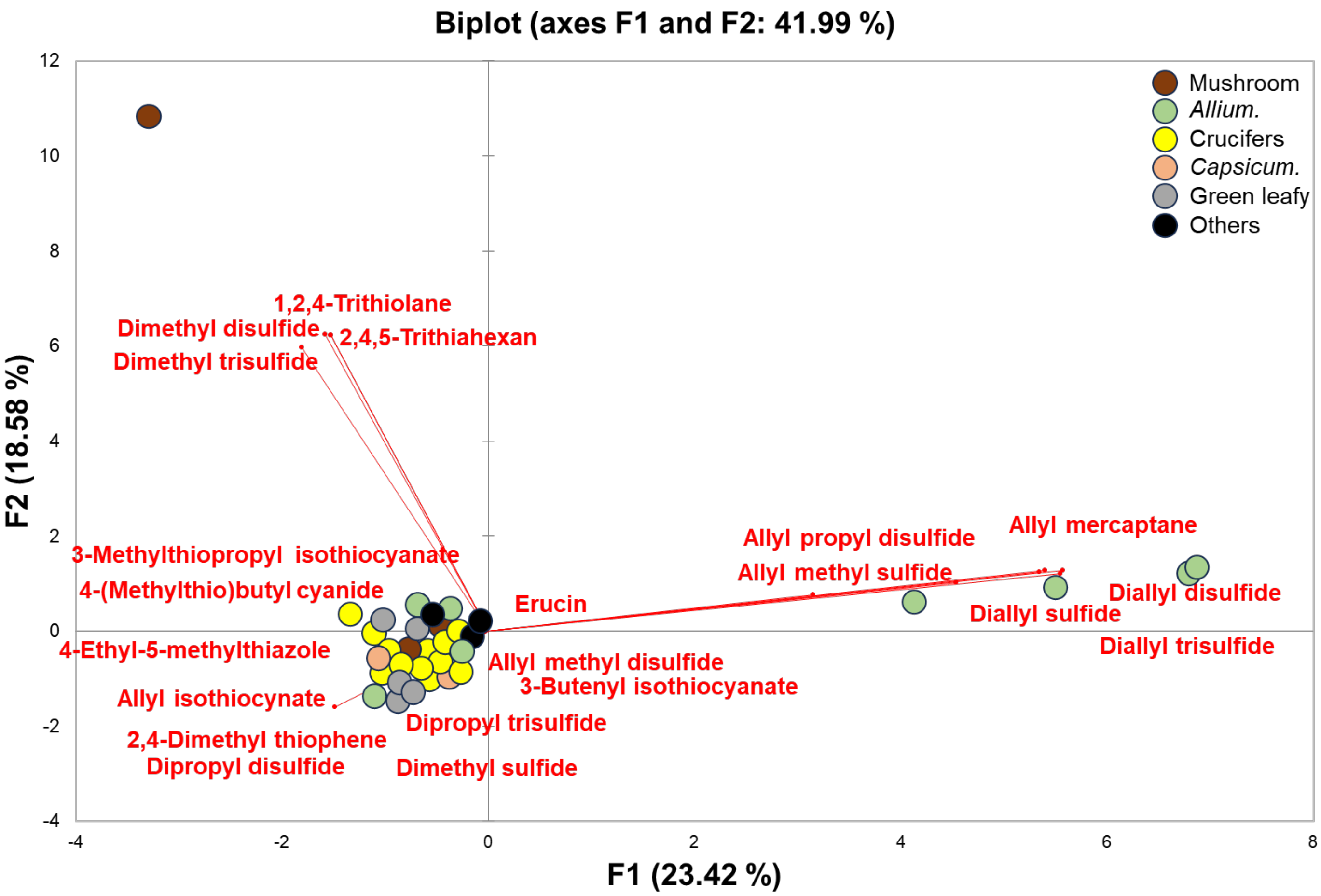

Supplementary Figure 3. Principal Component Analysis (PCA) of vegetables with detected VOCs

1.2 Supplementary Tables

Supplementary Table 1. The detailed list of samples used in this study are attached with additional file (Excel type – Table S1 tab))

Supplementary Table 2. The concentrations (mg/100g) of volatile sulfur compounds in different types of vegetables are attached with additional file (Excel type – Table S2 tab)
